# Landmark-specific phenotypic heterogeneity in CRRT discontinuation timing: a multi-landmark target trial emulation using MIMIC-IV

**DOI:** 10.3389/fmed.2026.1918781

**Published:** 2026-10-14

**Authors:** Yuan Zhou, Xiaoyu Zhang, Jiaxiang Chen, Jiahui Xie, Yuetang Wang, Runwei Ma

**Affiliations:** 1 Department of Cardiac Surgery, Fuwai Yunnan Hospital, Chinese Academy of Medical Sciences/Affiliated Cardiovascular Hospital of Kunming Medical University, Kunming, Yunnan, China; 2 Cardiovascular Surgery Department, Fuwai Hospital, Chinese Academy of Medical Sciences and Peking Union Medical College, National Center for Cardiovascular Diseases, Beijing, China

**Keywords:** continuous renal replacement therapy, CRRT discontinuation timing, target trial emulation, inverse probability weighting, marginal structural model, clone-censor-weighting, phenotypic heterogeneity, landmark analysis

## Abstract

**Background:**

The optimal timing for discontinuing continuous renal replacement therapy (CRRT) in critically ill patients remains uncertain. Observational studies commonly simplify discontinuation timing into “early” vs. “delayed” categories, yet the same label may correspond to different clinical states at different stages of illness. We evaluated whether the clinical meaning of the early-discontinuation label and its association with 30-day mortality varied across landmarks and analytic frameworks.

**Methods:**

Using MIMIC-IV (2008–2019), we emulated a target trial among adults who were alive and still receiving CRRT 48 h after initiation. Early discontinuation was defined as stopping CRRT within 24 h after the 48-hour landmark, whereas delayed discontinuation was defined as continuing CRRT beyond 24 h. The primary outcome was 30-day all-cause mortality. Analyses included crude Cox regression, baseline IPTW-Cox, multi-landmark analyses at 48, 72, 96, and 120 h, a marginal structural model (MSM) using time-varying IPTW, and clone-censor-weighting (CCW) with stabilized censoring weights under a 24-hour grace period. Phenotype decomposition was also performed to assess the clinical composition of patients classified as having early discontinuation at different landmarks.

**Results:**

A total of 1,432 patients were included, of whom 369 (25.8%) met the definition of early discontinuation. Baseline IPTW-Cox suggested that early discontinuation was associated with higher 30-day mortality (HR 1.36, 95% CI 1.13–1.64; *P* = 0.001). Multi-landmark HRs ranged from 1.13 to 1.35 without a monotonic pattern; precision decreased at later landmarks, and the landmark-by-exposure interaction was not statistically significant. The MSM estimate was OR 1.16 (95% CI 0.82–1.63; *P* = 0.40), and the revised CCW estimate was HR 1.08 (95% CI 0.91–1.28; *P* = 0.38). Phenotype decomposition showed that sepsis prevalence increased from 55.6% at 48 h to 69.1% at 120 h, baseline vasopressor exposure increased from 55.8% to 71.9%, and urine output decreased from 818 mL to 524 mL.

**Conclusions:**

Baseline IPTW generated a significant signal suggesting harm from early CRRT discontinuation, but this was not reproduced in multi-landmark, MSM, or CCW analyses. Phenotype decomposition indicated that “early weaning” captures different clinical profiles at different time points rather than a stable, homogeneous exposure. Observational CRRT weaning studies should consider dynamic treatment strategies, time-varying causal methods, and clinically grounded outcome definitions.

## Introduction

Continuous renal replacement therapy (CRRT) is an important form of organ support for critically ill patients with acute kidney injury (AKI), and is particularly suitable for patients who are hemodynamically unstable, have difficult-to-manage fluid balance, or have persistent metabolic disturbances (1, 2). The incidence of AKI is high in the ICU, and a significant proportion of critically ill patients ultimately require renal replacement therapy (3). Once CRRT is initiated, the clinical team is soon confronted with another equally critical but less well-evidenced question: When is it safe to discontinue CRRT?.

This decision is not straightforward. If CRRT is discontinued too early, the patient's recovering endogenous renal function may struggle to maintain volume, electrolyte, and acid-base balance, thereby increasing the risk of RRT reinitiation, volume rebound, or metabolic imbalance (4, 5). Conversely, delayed weaning may prolong vascular access exposure, increasing the risk of catheter-related infections, bleeding, thrombosis, and resource consumption (6, 7). However, current KDIGO guidelines recommend discontinuation only when RRT is “no longer required”—that is, when endogenous renal function has recovered sufficiently to meet the patient's needs, or when continued RRT no longer aligns with therapeutic goals (8). While this principle is clinically sound, it does not provide directly actionable weaning thresholds. Recent reviews and consensus statements also note that although urine output, creatinine clearance, hemodynamic stability, fluid balance, and disease severity have been repeatedly reported as factors associated with successful CRRT weaning, there is currently a lack of unified, validated, and widely applicable CRRT weaning criteria (9, 10).

Accordingly, the key evidence gap is not only the absence of an “optimal discontinuation time,” but also whether a fixed “early vs. delayed” timing label retains comparable clinical meaning when applied at different decision points. Previous studies have focused primarily on predicting success or failure after CRRT discontinuation, with few systematically examining whether the same binary label identifies comparable populations at different stages of illness (11). In real-world ICUs, CRRT discontinuation is a dynamic decision informed by renal recovery, hemodynamic status, net fluid goals, solute control, circuit status, treatment goals, and clinical judgment (12, 13). Thus, patients classified as “early” may include those with rapid renal recovery as well as those who stop CRRT because of changing treatment goals, failure of supportive care, or end-of-life decisions. Collapsing these distinct situations into a single binary label may obscure substantial clinical heterogeneity.

From a methodological perspective, CRRT discontinuation studies face at least three challenges. First, while a landmark design can reduce immortal time bias, it does not guarantee that “early discontinuation” at different landmark time points represents the same patient state. Patients classified as “early discontinuation” 48 h after CRRT initiation may have entirely different disease trajectories and physiological reserves compared to those who enter the same exposure category only after 120 h. Second, decisions to discontinue CRRT rely heavily on time-varying clinical information. For example, the previous day's urine output, mean arterial pressure (MAP), vasopressor requirements, and fluid balance can influence whether CRRT is discontinued on a given day; conversely, continuing or discontinuing CRRT on a given day may alter subsequent urine output, volume status, and metabolic environment (14). Such treatment-confounding feedback is difficult to adequately address using traditional regression models or propensity score methods based solely on baseline covariates (15). Finally, if the study objective is to evaluate a feasible discontinuation strategy rather than merely describing actual treatment choices, it is necessary to clarify how target trials, strategy adherence, imputed missing data, and time-varying confounders will be handled (16).

Given the above issues, this study employed a target trial emulation framework and utilized the MIMIC-IV database to assess the stability of the timing of CRRT weaning as a treatment exposure. We first compared the association between early and delayed weaning and 30-day mortality at the 48-hour landmark; subsequently, we used sequential landmark analysis to examine whether this association shifted as the time landmark changed; furthermore, we employed marginal structural models and the clone-censor-weighting method to address time-varying confounders and protocol adherence; Finally, we introduced phenotypic decomposition to describe whether the clinical characteristics of patients classified as “early weaning” underwent systematic changes at different landmark time points. Through this analytical chain, we aim to answer a question more fundamental than “whether early or late weaning is better”: in observational CRRT studies, is “early weaning” itself a stable, homogeneous, and causally comparable treatment exposure?.

## 2 Methods

### 2.1 Study design and target trial emulation framework

We used a target trial emulation framework to compare two CRRT discontinuation strategies in MIMIC-IV observational data (17, 37), prospectively aligning eligibility criteria, time zero, treatment strategies, follow-up, outcomes, and statistical analyses (Supplementary Table 1). The primary contrast compared CRRT discontinuation within 24 h after the 48-hour landmark with continuation beyond 24 h. The different analytic approaches were treated as addressing related but non-identical research questions rather than as repeated corrections of a single “true effect”.

### 2.2 Data sources and construction of CRRT treatment segments

MIMIC-IV v2.2 is a de-identified electronic health record database covering ICU admissions at Beth Israel Deaconess Medical Center between 2008 and 2019 (18); we drew on three of its modules—mimiciv_hosp, mimiciv_icu, and mimiciv_derived. As the data are fully de-identified, the study qualified for exemption from additional ethical review. CRRT episodes were identified from the mimiciv_derived.crrt table. Because filter changes, brief inter-unit transfers, or technical interruptions can artificially fragment a single course of treatment, consecutive CRRT records within the same ICU stay separated by 6 h or less were merged into one episode; for patients with more than one CRRT episode, only the first was retained for analysis.

### 2.3 Study population

Eligible patients were adults (age ≥18 years) who received CRRT during an ICU admission, with a first CRRT episode lasting at least 24 h. We excluded those with end-stage renal disease maintained on long-term dialysis, those receiving palliative care, and patients younger than 18 years. Time zero was set at 48 h after CRRT initiation, and only patients alive and still receiving CRRT at this landmark entered the primary analysis cohort. By excluding patients who died or discontinued CRRT before time zero, this landmark design guards against immortal time bias in the exposure comparison (19).

### 2.4 Exposure and outcome definitions

The primary exposure was residual CRRT duration after the 48-hour landmark. Patients were classified into an early-discontinuation group (residual duration ≤24 h, corresponding to a total CRRT duration of 48–72 h) and a delayed-discontinuation group (residual duration >24 h, corresponding to a total CRRT duration >72 h).

The 24-hour threshold was prespecified as a pragmatic grace period rather than as a validated clinical cutoff for CRRT liberation. This interval was chosen to distinguish near-term discontinuation after the landmark from continued CRRT support while preserving a clinically interpretable observation window. Previous reviews and consensus work have highlighted the absence of standardized CRRT liberation timing criteria and substantial heterogeneity in discontinuation definitions across studies (5, 14). We therefore examined alternative thresholds of ≤12, ≤36, and ≤48 h in sensitivity analyses. Patients who died while still receiving CRRT before any discontinuation event were treated as having experienced the outcome and were not reclassified as early discontinuation because treatment ended with death. If CRRT was discontinued within the 24-hour window and death occurred subsequently, exposure assignment remained early discontinuation. The CCW analysis used the same 24-hour grace period; artificial censoring was triggered only when the observed treatment path deviated from the assigned strategy, whereas death was always treated as an outcome. The primary outcome was 30-day all-cause mortality. Secondary outcomes included 60-day mortality, strict successful CRRT liberation, 28-day ICU-free days, and RRT dependence at hospital discharge.

### 2.5 Covariates and missing data handling

Fourteen baseline covariates were selected *a priori* based on clinical knowledge and a directed acyclic graph (Supplementary Figure 1). These variables were assessed within the 48-hour window before time zero and included age, sex, heart failure, chronic obstructive pulmonary disease, diabetes, liver disease, sepsis, SOFA score, minimum mean arterial pressure, baseline serum creatinine, 48-hour urine output, 24-hour net fluid balance, mechanical ventilation status, and vasopressor use. For these 14 baseline covariates used in the IPTW model, complete observations were available for 1,204 of 1,432 patients (84.1%) (Supplementary Table 2). Missing values were handled using multiple imputation by chained equations with predictive mean matching, generating 20 imputed datasets (20). A separate set of derived longitudinal variables was used in trajectory analyses, marginal structural modeling, and sensitivity analyses. These variables had higher missing proportions, partly because death or discharge precluded measurement in later assessment windows (Supplementary Table 2A). To reduce bias related to informative missingness, the imputation model included all baseline covariates, exposure status, outcome status, and the Nelson-Aalen cumulative hazard estimator. Imputation diagnostics were assessed using trace plots and density plots (Supplementary Figure 2).

### 2.6 Statistical analysis

All analyses were performed in R version 4.5.2. Each method targeted a related but non-identical estimand (Supplementary Table 3): crude Cox regression described the unadjusted association; baseline IPTW-Cox estimated a landmark-specific marginal contrast at the 48-hour landmark after balancing measured baseline covariates; multi-landmark analyses estimated corresponding landmark-specific contrasts within changing risk sets; the MSM reported a marginal contrast as a discrete-time OR after adjustment for measured time-varying confounding; and CCW estimated a marginal per-protocol contrast of adherence to the prespecified early or delayed strategy within the 24-hour grace period. Accordingly, numerical differences across models were interpreted as triangulation across distinct estimands and risk sets rather than as sequential correction of a single treatment effect.

#### 2.6.1 Crude cox regression

An unadjusted Cox proportional hazards model, including only the early-discontinuation indicator and using cluster-robust standard errors, served as the reference analysis.

#### 2.6.2 Baseline IPTW-cox

Stabilized inverse probability of treatment weighting (IPTW) was used to address baseline confounding (21), with propensity scores estimated from a logistic model containing all 14 baseline covariates. Post-weighting covariate balance was assessed by standardized mean differences (SMD), with SMD <0.1 taken as adequate (Supplementary Figure 5a). Each of the 20 imputed datasets was analyzed separately, and the resulting estimates were pooled using Rubin's rules (22).

Multi-landmark analysis. Time zero was sequentially reset at 48, 72, 96, and 120 h after CRRT initiation; at each landmark, patients alive and still receiving CRRT were retained, residual CRRT duration was recalculated, and the IPTW-Cox analysis was repeated. We assessed temporal stability of the exposure–outcome association both by inspecting HR variation across landmarks and by formally testing the exposure×landmark interaction.

#### 2.6.3 Marginal structural model (MSM)

To account for time-varying confounding, data were restructured into patient-day format (23). The time-varying exposure was daily CRRT status, and the time-varying confounders were the previous day's (lagged) urine output, MAP, and vasopressor use. Stabilized weights, calculated as the product of treatment and censoring weights and truncated at the 1st and 99th percentiles (Supplementary Figure 3), were entered into a pooled logistic regression with cluster-robust standard errors to estimate the MSM. The positivity assumption was examined through the distribution of predicted treatment probabilities, and sensitivity analyses explored multiple weight-truncation thresholds.

#### 2.6.4 Clone-censor-weighting (CCW)

At the 48-hour landmark, each eligible patient was cloned into two copies, one assigned to the early-discontinuation strategy and the other to the delayed-discontinuation strategy. A 24-hour grace period was allowed for strategy classification. Clones were artificially censored when their observed treatment path became incompatible with the assigned strategy. Stabilized inverse probability of censoring weights were estimated using baseline clinical covariates measured before the landmark, and weights were truncated at the 1st and 99th percentiles in the primary analysis. A weighted Cox model with patient-level robust standard errors was then used to estimate the strategy-level association with 30-day mortality. Weight distributions, effective sample size, and alternative truncation thresholds were evaluated as diagnostics and sensitivity analyses.

#### 2.6.5 Phenotype decomposition

To determine whether the “early weaning” label identifies comparable patient populations across landmarks, we compared clinical characteristics—sepsis prevalence, vasopressor use, urine output, and SOFA score—among patients so classified at 48, 72, 96, and 120 h. Logistic regression was used to estimate the change in the odds of baseline sepsis and baseline vasopressor exposure per 24-hour later landmark, with ORs and 95% CIs reported. Urine output was summarized descriptively because its pattern was non-monotonic.

#### 2.6.6 Sensitivity analyses

Three sensitivity analyses were conducted: excluding patients who died within 24 h of CRRT discontinuation, to limit contamination from end-of-life weaning; redefining early weaning using alternative thresholds (≤12, ≤36, and ≤48 h); and calculating E-values to quantify the potential impact of unmeasured confounding (24).

#### 2.6.7 Multi-state model

Clinical trajectories following CRRT discontinuation were further characterized using a multi-state model, beginning from the state “on CRRT” and transitioning to “CRRT discontinued,” “strict successful liberation,” “RRT restarted,” “death after CRRT discontinuation,” or “death without CRRT discontinuation.” These endpoint states were defined as mutually exclusive, explicitly distinguishing CRRT discontinuation from successful liberation; the full model structure is presented in Supplementary Figure 4.

## 3 Results

### 3.1 Study population and baseline characteristics

From 3,597 ICU stays involving CRRT, 3,519 entered the preliminary cohort after excluding 78 patients with end-stage renal disease (Figure 1). At the 48-hour landmark, 1,432 patients (40.7%) remained alive and on CRRT and were retained for the primary analysis cohort: 369 (25.8%) met criteria for early discontinuation and 1,063 (74.2%) for delayed discontinuation. Median residual CRRT duration was 14.0 h (IQR 6.0–24.0) in the early group, compared with 83.2 h (IQR 47.0–138.4) in the delayed group. Mean residual duration was 86.3 h (SD 92.6). Overall 30-day mortality was 47.4% (679/1,432), with comparable rates in the early (48.2%, 178/369) and delayed (47.1%, 501/1,063) groups.

**Figure 1:**
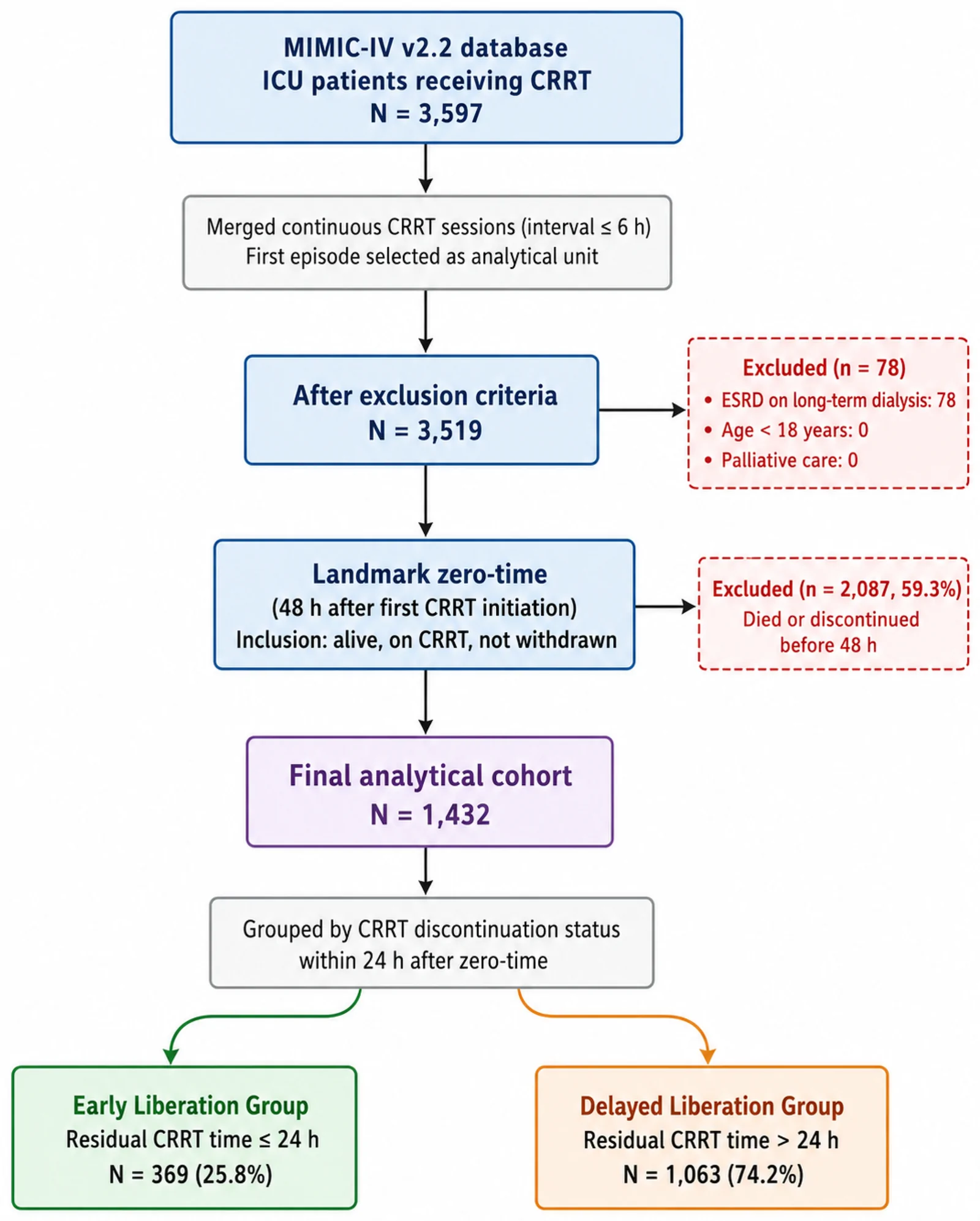
Patient screening flowchart.

Before weighting, patients undergoing early discontinuation presented with a milder disease profile—lower SOFA scores (11.4 ± 3.9 vs. 12.3 ± 3.7),less frequent baseline vasopressor use (55.8% vs. 70.6%), and lower sepsis prevalence (55.6% vs. 65.6%) (Table 1). IPTW weighting achieved excellent covariate balance, with standardized mean differences for all 14 baseline covariates falling below 0.1 (Supplementary Figure 5b); the largest pre-weighting imbalance, for SOFA score (SMD 0.24), was reduced to 0.024. Stabilized weights were centered near unity (mean 1.00; range 0.60–1.61), yielding an effective sample size of 1,394.

**Table 1:** Baseline characteristics before and after IPTW weighting.

	Unweighted				IPTW Weighted	
Characteristic	Overall *N* = 1,432	Delayed(>24 h) *N* = 1,063	Early(<=24 h) *N* = 369	*P* value	Delayed(>24 h) *N* = 1,063	Early(<=24 h) *N* = 369
anchor_age	59.7 (14.6)	59.6 (14.6)	60.1 (14.7)	0.583	59.7	59.8
Gender						
Male	869 (61%)	645 (61%)	224 (61%)	1.000	61%	61%
Heart failure	600 (42%)	434 (41%)	166 (45%)	0.166	41.8%	41.3%
COPD	808 (56%)	587 (55%)	221 (60%)	0.117	56.4%	55.8%
Diabetes	574 (40%)	418 (39%)	156 (42%)	0.322	40.3%	41.9%
Liver disease	565 (39%)	427 (40%)	138 (37%)	0.346	39.6%	40.7%
Sepsis	902 (63%)	697 (65.6%)	205 (55.6%)	<0.001	63.2%	64.2%
SOFA score	12.1 (3.8)	12.3 (3.7)	11.4 (3.9)	<0.001	12.1	12.1
Minimum MAP (mmHg)	57.6 (11.4)	57.5 (10.1)	57.6 (14.4)	0.881	57.5	57.3
Creatinine (mg/dL)	1.8 (0.8)	1.8 (0.8)	1.8 (0.9)	0.568	1.8	1.8
48 h urine output (mL)	694.5 (2,208.2)	652.2 (1,952.2)	818.0 (2,825.9)	0.334	640.6	634.8
24 h net fluid balance (mL)	13,764.5 (16,077.7)	14,312.7 (16,613.1)	12,207.4 (14,356.6)	0.021	13,688.1	13,939.6
Mechanical ventilation	1,273 (89%)	950 (89%)	323 (88%)	0.351	88.9%	89.3%
Vasopressor use	957 (66.8%)	751 (70.6%)	206 (55.8%)	<0.001	66.9%	66.3%
Exposure and outcome						
Residual CRRT (hours)	86.3 (92.6)	111.4 (95.3)	14.0 (6.9)	<0.001	110.2 (83.2)	13.9
30-day mortality	679 (47%)	501 (47%)	178 (48%)	0.714	46.3%	52.0%

Data are presented as means (standard deviations) or percentages. The 14 baseline covariates were assessed within a 48-hour window prior to the zero time point. After IPTW weighting, the balance of covariates between the two groups was excellent (all standardized mean differences <0.1; see Supplementary Figure 5a). The standardized mean difference (SMD) for the SOFA score was highest before weighting (0.24) and decreased to 0.024 after weighting. Categorical variables were reported as weighted percentages after weighting (case counts were not reported due to continuous weights).

SOFA, sequential organ failure assessment; MAP, mean arterial pressure; COPD, chronic obstructive pulmonary disease; CRRT, continuous renal replacement therapy.

### 3.2 The main associations of the 48-hour landmark

At the 48-hour landmark, MI-IPTW–pooled Cox regression yielded a hazard ratio of 1.36 for early relative to delayed CRRT discontinuation (95% CI 1.13–1.64; *P* = 0.001) (Table 2). All 20 imputed datasets agreed on the direction of effect, with HRs uniformly above 1. The weighted Kaplan–Meier curves diverged shortly after time zero and remained separated throughout follow-up, with the early discontinuation group showing persistently lower survival probability (Figure 2).

**Table 2:** Summary of major analyses and sensitivity analyses.

Analysis	Effect measure	Estimate(95%CI)	*P* value
Main Analysis			
MI-IPTW combined with Cox (48 h Landmark)	HR	1.36 (1.13–1.64)	0.001
crude Cox (unadjusted)	HR	1.15 (0.96–1.38)	0.15
Landmark Drift Analysis			
48h	HR	1.32 (1.10–1.59)	0.003
72h	HR	1.14 (0.91–1.42)	0.25
96h	HR	1.35 (1.05–1.74)	0.02
120h	HR	1.13 (0.84–1.52)	0.81
Time-varying Systems and Strategy Analysis			
MSM (Time-Varying IPTW, with merged logistic regression)	OR	1.16 (0.82–1.63)	0.40
CCW	HR	1.08 (0.91–1.28)	0.38
RMST	Days	3.2 (Delayed−Early)	*P* < 0.001
Secondary Outcomes			
Death within 60 days	HR	1.25 (1.04–1.49)	0.017
Strict successful CRRT liberation	OR	0.98 (0.75–1.26)	0.86
28-day ICU-free days	*β* (days)	1.08 (0.10–2.06)	0.031
Discharge from hospital with dependence on continuous renal replacement therapy (CRRT)	OR	0.93 (0.72–1.18)	0.54
sensitivity analysis			
after excluding potential terminal CRRT discontinuation	HR	1.38 (1.13–1.68)	0.001
E-value(48 h HR = 1.32)			
point estimation	—	1.97	—
CI lower limit	—	1.43	—

**Figure 2:**
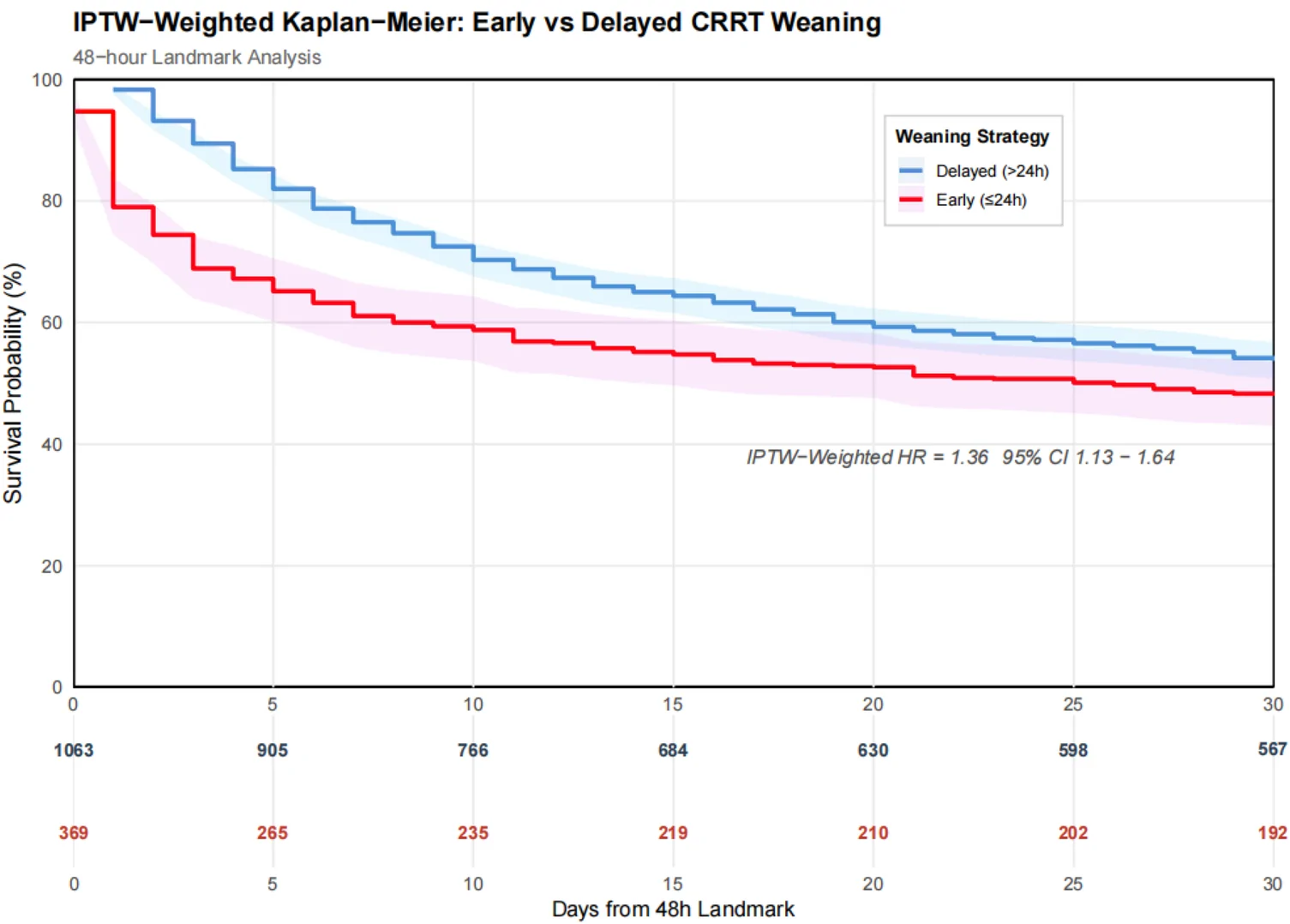
IPTW-weighted survival curves by CRRT discontinuation strategy. Weighted Kaplan–Meier curves comparing early discontinuation (residual CRRT ≤24 h after the 48-hour landmark) with delayed discontinuation (>24 h). The risk table shows numbers at risk after the landmark. CRRT, continuous renal replacement therapy; IPTW, inverse probability of treatment weighting.

### 3.3 Landmark drift analysis

Across the four sequential landmarks, effect estimates fluctuated without a clear monotonic pattern (Figure 3, Table 2): 1.32 (95% CI 1.10–1.59) at 48 h, 1.14 (0.91–1.42) at 72 h, 1.35 (1.05–1.74) at 96 h, and 1.13 (0.84–1.52) at 120 h. The global landmark-by-exposure interaction was not statistically significant (*P* > 0.05), although the analyzable risk set decreased substantially over time, from 1,432 to 595 patients (Supplementary Table 5).

**Figure 3:**
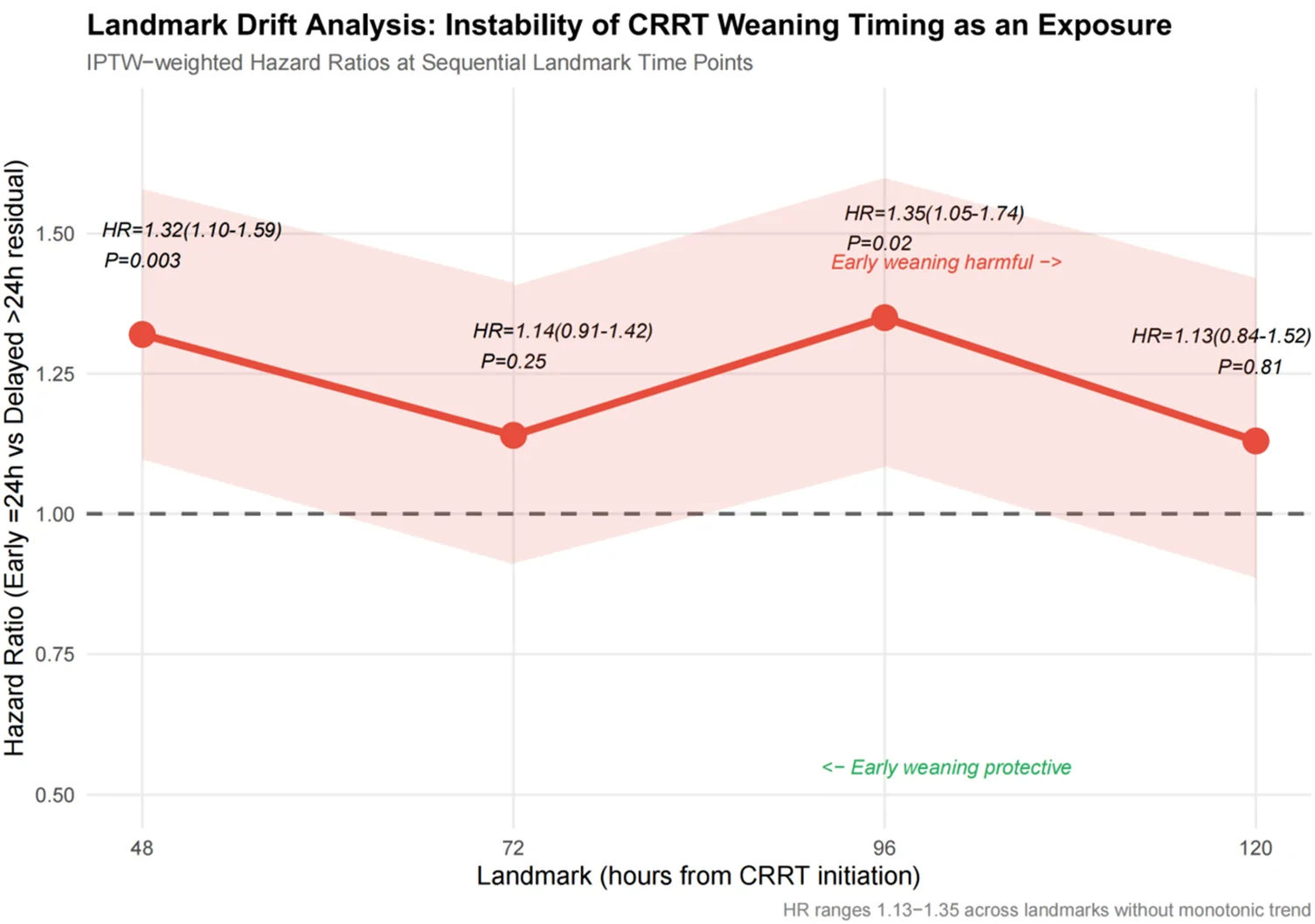
Landmark-specific estimates for early versus delayed CRRT discontinuation. IPTW-weighted hazard ratios were estimated at sequential landmarks 48, 72, 96, and 120 h after CRRT initiation. Points represent hazard ratios and error bars represent 95% confidence intervals. The dashed line indicates the null value. CRRT, continuous renal replacement therapy; IPTW, inverse probability of treatment weighting.

### 3.4 Temporal variation and strategy analysis

In the MSM, stabilized weights averaged 0.88 (truncated range 0.25–5.67), and substantial overlap in predicted treatment probabilities across exposure strata supported the positivity assumption (Supplementary Figure 3). The resulting estimate—OR 1.16 (95% CI 0.82–1.63; *P* = 0.40)—remained stable across weight-truncation thresholds (OR range 1.16–1.20). Window-specific missingness increased over time, particularly for lagged urine output; the proportion of records with all three time-varying covariates available declined from 66.7% at 48 h to 48.5% at 120 h (Supplementary Table 2A).

In the CCW analysis, 74.2% of Clone A (assigned to the early strategy) and 25.8% of Clone B (assigned to the delayed strategy) were artificially censored for protocol deviation. The full CCW model produced a per-protocol HR of 1.08 (95% CI 0.91–1.28; *P* = 0.38), with a reasonable IPCW weight distribution (effective sample size 1,432). After IPTW weighting, the 30-day restricted mean survival time was 17.5 days (SE 0.68) in the early discontinuation group vs. 20.7 days (SE 0.35) in the delayed group, a 3.2-day survival advantage favoring delayed discontinuation. Taken together, point estimates from the five analytic approaches moved progressively toward the null as each successive method addressed an additional source of bias: crude Cox 1.15 → baseline IPTW 1.36 → MSM 1.16 → CCW 1.08 (Figure 4, Table 2).

**Figure 4:**
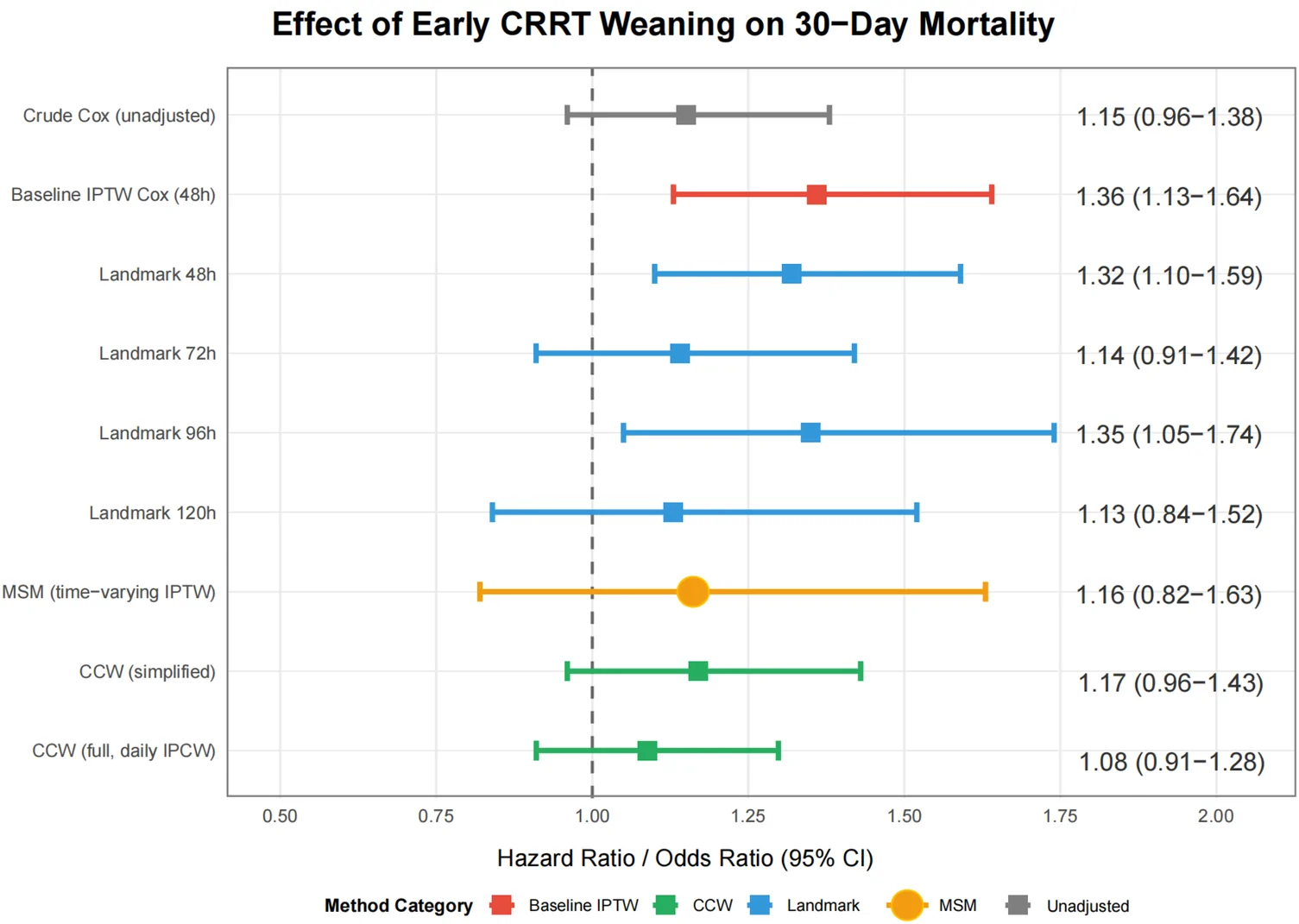
Effect estimates across complementary analytic approaches. The forest plot displays estimates from crude Cox regression, baseline IPTW-Cox models, multi-landmark analyses, the marginal structural model, and clone-censor-weighting. Squares denote hazard ratios (HRs) from Cox-based and CCW analyses, whereas circles denote the discrete-time odds ratio (OR) from the MSM. Given the 30-day mortality rate of 47.4% in this cohort, ORs and HRs should not be interpreted as numerically interchangeable. CCW, clone-censor-weighting; CI, confidence interval; IPTW, inverse probability of treatment weighting; MSM, marginal structural model.

### 3.5 Phenotypic decomposition

Across the four landmarks, the clinical composition of patients classified as early CRRT discontinuation differed systematically (Supplementary Table 6, Panel A; Figure 5). Sepsis prevalence increased from 55.6% at the 48-hour landmark to 69.1% at 120 h. In formal trend analyses (Supplementary Table 6, Panel B), each 24-hour later landmark was associated with 21% higher odds of baseline sepsis (OR 1.21, 95% CI 1.07–1.37; *P* = 0.002). Baseline vasopressor exposure was also more prevalent among patients classified as early discontinuation at later landmarks, increasing from 55.8% at 48 h to approximately 72% at the 96- and 120-hour landmarks; the corresponding OR per 24-hour later landmark was 1.32 (95% CI 1.16–1.50; *P* < 0.001; Supplementary Table 6, Panel B). Urine output showed a non-monotonic pattern, increasing from 818 mL at 48 h to 892 mL at 72 h before declining to 524 mL at 120 h, whereas baseline SOFA score increased from 11.4 to 12.3. These findings indicate that the same operational definition of early discontinuation identified clinically different patient populations depending on the landmark at which it was applied.

**Figure 5:**
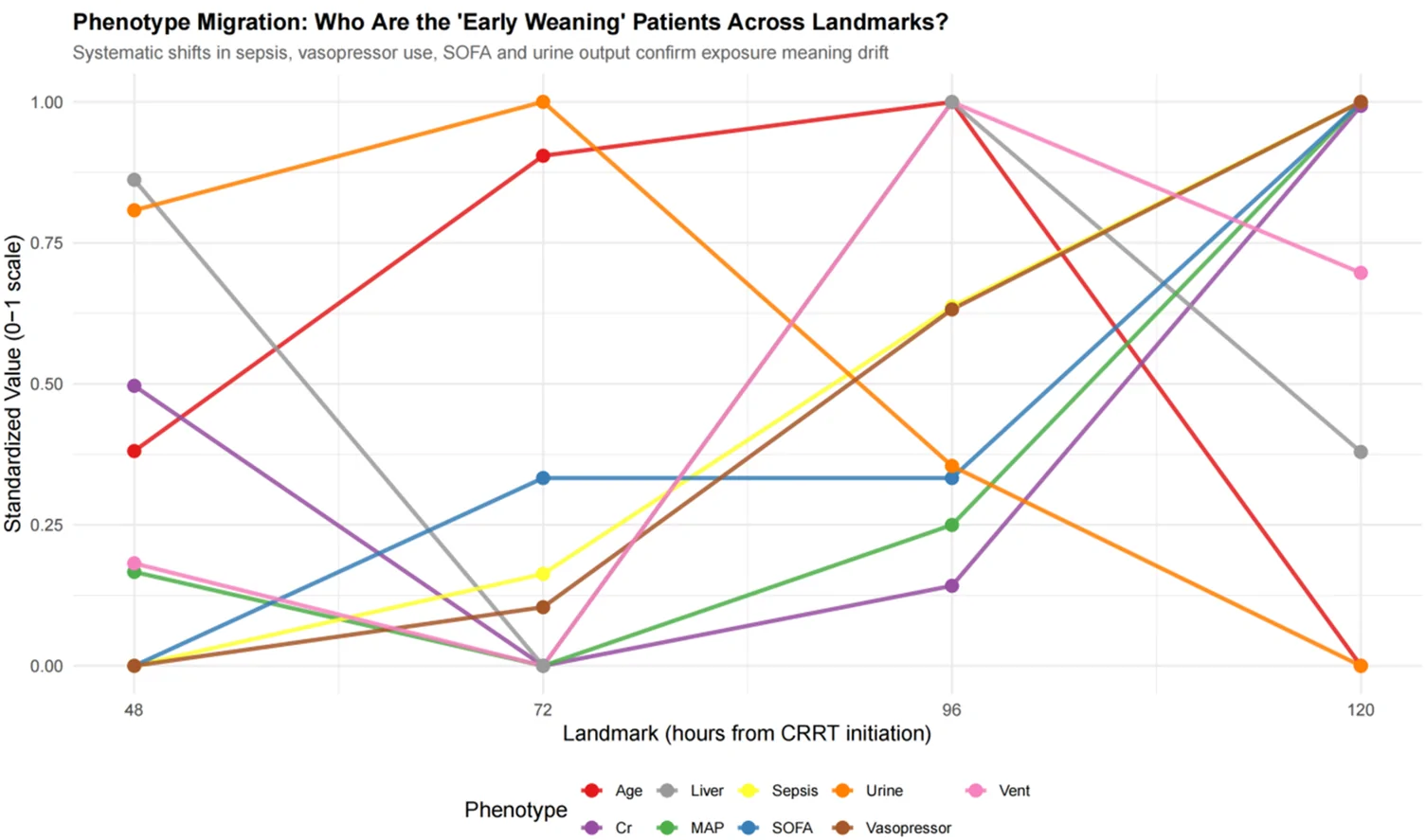
Phenotypic shifts among patients classified as early CRRT discontinuation across landmarks. Standardized trajectories of selected clinical characteristics among patients classified as early discontinuation at 48, 72, 96, and 120 h. The plot illustrates changes in the clinical profile of the early discontinuation group across landmark times. CRRT, continuous renal replacement therapy; MAP, mean arterial pressure; SOFA, Sequential Organ Failure Assessment.

### 3.6 Validation of landmark drift after phenotypic restriction

Landmark-dependent variation persisted after restricting to a more clinically homogeneous subgroup. Among patients with a SOFA score of 8–14 (*n* = 868), HRs ranged from 0.98 to 1.27 (Supplementary Figure 6, Supplementary Table 7)—a spread comparable to that of the full cohort (1.13–1.35)—indicating that phenotype restriction did not meaningfully attenuate landmark drift. Under the more stringent restriction of SOFA 8–14 without sepsis (*n* = 326), reduced event counts produced correspondingly wide confidence intervals.

### 3.7 Secondary outcomes and sensitivity analysis

Secondary outcomes were broadly consistent with the primary findings. The hazard ratio for 60-day mortality was 1.25 (95% CI 1.04–1.49). Early discontinuation was not associated with strict successful CRRT liberation (OR 0.98, 95% CI 0.75–1.26; *P* = 0.86) or with RRT dependence at discharge (OR 0.93, 95% CI 0.72–1.18; *P* = 0.54), although 28-day ICU-free days showed a small increase (*β*=1.08 days, 95% CI 0.10–2.06; *P* = 0.031).

Ninety-four patients (6.6%) died within 24 h of CRRT discontinuation and were classified as potential end-of-life weaning; these events were distributed similarly between groups (6.0% vs. 6.8%). Excluding these patients yielded a slightly higher hazard ratio of 1.38 (95% CI 1.13–1.68). The pattern of HR fluctuation across landmarks persisted under alternative exposure thresholds of ≤12, ≤36, and ≤48 h (Supplementary Figure 7). For the 48-hour HR of 1.32, the E-value was 1.97 for the point estimate and 1.43 for the lower confidence limit (Table 2).

### 3.8 Multistate model and physiological trajectories

The multistate model showed that 448 patients (31.3%) discontinued CRRT within 30 days. Among those who stopped, 159 (35.5%) achieved strict successful liberation, 129 (28.8%) restarted RRT within 72 h, and 160 (35.7%) died after discontinuation. A further 472 patients (33.0%) died without ever discontinuing CRRT, and 512 (35.8%) remained alive on CRRT at 30 days (Supplementary Figure 4). These results indicate that CRRT discontinuation is an intermediate clinical state rather than a definitive marker of recovery. Physiological trajectories also differed between the early and delayed discontinuation groups (Supplementary Figure 8). At 24 h after the landmark, urine output was higher in the early group than in the delayed group (152.6 vs. 99.3 mL/6 h), and this difference persisted at 96 h (305.2 vs. 136.3 mL/6 h). Landmark lactate was slightly lower in the early group (3.07 vs. 3.54 mmol/L). These trajectory patterns suggest that observed discontinuation timing was closely linked to evolving clinical recovery, rather than representing an isolated treatment exposure.

## 4 Discussion

The main finding of this study is not that early CRRT discontinuation necessarily increases mortality, but that estimates of the association between early discontinuation and mortality were not consistent across landmarks and analytic frameworks. The 48-hour baseline IPTW analysis suggested a higher mortality risk, whereas the multi-landmark analyses, MSM, and revised CCW did not provide consistent statistical evidence of harm. More directly, phenotype decomposition showed systematic changes in sepsis burden, baseline vasopressor exposure, SOFA score, and urine output among patients captured by the same “early discontinuation” label as the landmark shifted from 48 to 120 h. These findings support landmark-specific phenotypic heterogeneity rather than treating “early discontinuation” itself as a directly measurable unstable treatment exposure.

The central finding of this study is therefore not that early CRRT discontinuation is intrinsically harmful, but that the label “early” may have unstable clinical meaning in observational data. If one relied only on the baseline IPTW analysis, the conclusion would be that early discontinuation increases mortality. Yet the sequential landmark analysis showed numerically variable hazard ratios between 1.13 and 1.35 without a monotonic pattern; after incorporating daily CRRT status and time-updated urine output, mean arterial pressure, and vasopressor use in the marginal structural model, the estimate moved toward the null and was no longer statistically significant. The full clone-censor-weighting analysis likewise did not support a statistically significant strategy effect. Thus, the baseline IPTW signal may reflect residual time-varying confounding, heterogeneity in weaning readiness, or selection processes related to evolving clinical status. It should not be interpreted as a stable causal effect of an early discontinuation strategy.

This interpretation is consistent with the bedside logic of CRRT weaning. CRRT discontinuation is rarely a single discrete therapeutic act; it is a clinical decision that integrates renal recovery, hemodynamic stability, solute control, fluid balance, respiratory status, vasopressor requirements, treatment goals, and circuit or filter status (4). A recent Delphi consensus also emphasized that widely applicable CRRT weaning criteria remain lacking and that discontinuation should be understood as a recovery-oriented clinical process rather than a simple time threshold (25). Prior studies and systematic reviews have identified urine output, creatinine clearance, disease severity, vasopressor exposure, and fluid balance as factors associated with successful CRRT liberation (26, 27). Our results extend this evidence by showing that these variables do more than confound the exposure-outcome relationship; they also shape what the exposure label “early discontinuation” means at any given time point.

Phenotype decomposition provides a clinical explanation for this landmark-specific heterogeneity. At the 48-hour landmark, patients classified as early discontinuation more closely resembled rapidly recovering patients, with lower sepsis burden, less vasopressor use, and higher urine output. By 120 h, however, patients carrying the same label had a more severe clinical phenotype, with higher sepsis prevalence, greater vasopressor exposure, higher SOFA scores, and lower urine output. Thus, “early discontinuation” at 48 h and at 120 h may represent different clinical decisions. The former may more often reflect recovery-driven discontinuation, whereas the latter may involve delayed recovery, treatment limitation, inability to continue extracorporeal support, circuit-related issues, or other complex clinical pathways. Although the exposure label itself is unchanged, the corresponding patient population and decision context shift over time, limiting the interpretability of a single fixed effect estimate.

This study also fits within the broader literature on RRT timing in critical illness. The AKIKI, IDEAL-ICU, and STARRT-AKI trials, together with individual patient-data meta-analyses, have shown that accelerated or early RRT initiation does not consistently improve survival in critically ill patients with AKI when urgent indications are absent (28–31). These studies shifted the focus from isolated biochemical thresholds toward treatment strategies. CRRT discontinuation is even more complex because discontinuation occurs after the patient has already responded, or failed to respond, to prior support. Urine output, lactate, fluid balance, vasopressor requirements, and hemodynamic status influence the decision to stop CRRT; in turn, stopping or continuing CRRT can alter subsequent volume status, metabolic control, and clinical trajectory. This treatment-confounder feedback is difficult to address adequately using conventional regression or baseline propensity score methods (32).

The divergence between baseline IPTW and time-varying or strategy-based analyses is therefore methodologically informative. Baseline IPTW can balance measured covariates before the landmark but cannot account for subsequent changes in urine output, blood pressure, vasopressor exposure, fluid balance, and clinical response. Marginal structural models address treatment-confounder feedback by incorporating time-updated covariates. Clone-censor-weighting compares adherence to prespecified discontinuation strategies by cloning patients into both strategies, censoring clones when they deviate from the assigned strategy, and applying inverse probability of censoring weights. In the present study, the simplified and full CCW estimates were empirically similar, suggesting that the available time-updated covariates did not fully explain strategy deviation in this dataset. This should not be interpreted as a failure of the method; rather, it highlights that unmeasured factors such as treatment limitations, filter clotting, net ultrafiltration goals, diuretic strategies, staffing patterns, and clinician preference may still influence discontinuation decisions and outcomes.

Another important finding is that CRRT discontinuation should not be equated with successful liberation. In the multistate trajectory, 31.3% of patients discontinued CRRT within 30 days. Among those who stopped, only 35.5% achieved strict successful liberation, whereas 28.8% required RRT reinitiation within 72 h and 35.7% died after discontinuation. A further 33.0% died without a CRRT discontinuation attempt. These findings underscore that CRRT discontinuation is a transitional state rather than a definitive endpoint. Previous studies have similarly suggested that failure to achieve sustained CRRT liberation is common and prognostically important (33, 34). Future studies should therefore consider process-sensitive outcomes, including RRT-free survival, MAKE30 or MAKE90, ICU-free days, and multistate transition probabilities, rather than relying solely on first discontinuation time or mortality (35).

This study offers several implications for future CRRT weaning research. First, discontinuation timing should be conceptualized as a dynamic treatment strategy rather than a fixed binary exposure. Second, landmark time is not a neutral technical choice; the same exposure definition may identify different patient phenotypes at different time points. Third, successful CRRT liberation should be distinguished from simple device discontinuation. Fourth, target-trial emulations should explicitly report eligibility criteria, treatment strategies, time zero, follow-up, causal contrasts, identification assumptions, adherence definitions, missing-data handling, and sensitivity analyses to improve interpretability and reproducibility (36).

### 4.1 Several limitations should be acknowledged

First, this study was based on a single-center ICU database, and CRRT initiation, discontinuation practices, ultrafiltration targets, and clinician thresholds may differ across institutions, limiting generalizability. Second, although multiple imputation, baseline IPTW, marginal structural modeling, and clone-censor-weighting were used to address measured confounding and time-related bias, residual confounding remains possible. In particular, time-varying adjustment was limited by the granularity and completeness of the electronic health record. Urine output, mean arterial pressure, and vasopressor use were available for longitudinal modeling, whereas other clinically relevant determinants of CRRT discontinuation—including dynamic fluid balance, creatinine-clearance trajectories, lactate kinetics, diuretic responsiveness, and changes in treatment goals—could not be incorporated consistently. Accordingly, the MSM and CCW estimates should be interpreted cautiously as they may remain affected by unmeasured treatment-confounder feedback. Third, CRRT treatment episodes were reconstructed from electronic health record data, and brief interruptions, filter changes, transfers, or documentation gaps may have introduced some exposure misclassification despite prespecified episode-merging rules. Fourth, the analytic approaches used in this study target related but non-identical estimands and report different effect measures. In particular, the Cox-based analyses and CCW report hazard ratios, whereas the discrete-time MSM reports an odds ratio. Given the relatively high 30-day mortality rate, these measures are not numerically interchangeable. Differences across models should therefore be interpreted as triangulation across complementary analytic frameworks rather than as sequential refinement of a single common effect estimate. Fifth, precision decreased as the eligible risk set became smaller at later landmarks. At 120 h, the HR was 1.13 (95% CI 0.84–1.52), and the non-significant landmark-by-exposure interaction should therefore be interpreted as an absence of statistically detectable interaction rather than evidence of effect homogeneity; moderate between-landmark heterogeneity may have remained undetected. Finally, longitudinal variables had substantial missingness, part of which was structurally related to death or discharge. Although survival information was incorporated into the imputation procedure, informative missingness cannot be completely eliminated. External validation in multicenter cohorts with standardized recording of CRRT timing, renal recovery, and treatment goals is therefore warranted.

## 5 Conclusion

This study demonstrates that the observational association between the timing of CRRT discontinuation and mortality varies depending on the landmark risk set and analytical framework employed. Phenotypic decomposition further reveals that patients classified as having “early discontinuation” at different time points exhibit systematically distinct clinical characteristics, supporting the concept of landmark-specific phenotypic heterogeneity and suggesting that the clinical implications of a fixed residual time threshold are time-dependent. Future research should prioritize evaluating well-defined dynamic discontinuation strategies and prospectively document the intention to discontinue CRRT, renal function recovery, fluid/ultrafiltration targets, and changes in treatment goals within multicenter datasets.

## Data Availability

The datasets presented in this study can be found in online repositories. The names of the repository/repositories and accession number(s) can be found below: The data underlying this study are publicly available from the MIMIC-IV database, which can be accessed through PhysioNet at: https://physionet.org/content/mimiciv/. The MIMIC-IV database is hosted on PhysioNet (https://physionet.org) and requires completion of a data use agreement and completion of a human subjects research training course (CITI Program). Upon approval, users can download the complete dataset. Database identifier: MIMIC-IV v2.2Repository: PhysioNet (https://physionet.org) DOI: https://doi.org/10.13026/6hk1-md68.
