# Supplementary material for: Landmark-specific phenotypic heterogeneity in CRRT discontinuation timing: a multi-landmark target trial emulation using MIMIC-IV
Source: Front Med (Lausanne). 2026 Oct 14;13:1918781. doi: 10.3389/fmed.2026.1918781 (PMC13713494; doi:10.3389/fmed.2026.1918781)
Supplement: Supplementary file 1 [file Table1.docx]

**Supplemental Instrument**

**Supplementary Table S1. Alignment of the Hypothetical Target Trial, Conventional Observational Design, and the Present Target-Trial Emulation**

| **Essential component** | **Hypothetical randomized trial** | **Conventional observational cohort study** | **Target-trial emulation in the present study** |
| --- | --- | --- | --- |
| Study population | Adult ICU patients with AKI who survived for at least 48 hours after first CRRT initiation and remained on CRRT at 48 hours | Adult ICU patients receiving CRRT, often classified according to the observed timing of discontinuation | Same as the hypothetical trial; eligible patients were identified from the MIMIC-IV CRRT records |
| Eligibility criteria | Age ≥18 years; first CRRT episode lasting ≥24 hours; alive and still receiving CRRT at the 48-hour time zero | Eligibility may vary; patients may be included without explicitly aligning eligibility, exposure classification, and time zero | Age ≥18 years; first CRRT episode lasting ≥24 hours; alive and still receiving CRRT at the 48-hour landmark |
| Exclusion criteria | End-stage kidney disease or long-term dialysis; palliative care; age <18 years | Exclusions may be applied, but time-related exclusions are often not explicitly linked to a defined time zero | Same as the hypothetical trial; ESRD/long-term dialysis and palliative care were identified using ICD-9/ICD-10 codes |
| Time zero | 48 hours after first CRRT initiation | Often undefined or implicitly set at ICU admission, CRRT initiation, or CRRT discontinuation, which may introduce immortal-time bias | 48 hours after first CRRT initiation; only patients alive and still on CRRT at this landmark were included |
| Treatment strategies | Early discontinuation: stop CRRT within 24 hours after time zero; delayed discontinuation: continue CRRT beyond 24 hours after time zero | Observed treatment duration is compared retrospectively, usually without a prespecified strategy or grace period | Early discontinuation was defined as residual CRRT time ≤24 hours after the 48-hour landmark; delayed discontinuation as residual CRRT time >24 hours |
| Treatment assignment | Random allocation to early or delayed discontinuation strategy | Determined by clinical status, clinician judgment, treatment goals, and evolving physiologic response | Not randomized; treatment selection was addressed using baseline IPTW, time-varying MSM, and CCW analyses |
| Primary outcome | 30-day all-cause mortality from time zero | Mortality is commonly assessed from ICU admission, CRRT initiation, or discontinuation, depending on study design | 30-day all-cause mortality from the 48-hour landmark |
| Secondary outcomes | 60-day mortality; successful CRRT liberation; ICU-free days; RRT dependence at discharge | Variable across studies; successful liberation and RRT restart may not be consistently distinguished | 60-day mortality; strict successful CRRT liberation; 28-day ICU-free days; RRT dependence at discharge |
| Causal contrast | Intention-to-treat effect of assignment to early versus delayed discontinuation strategy; per-protocol effect if adherence is enforced | Association between observed discontinuation timing and outcome; causal interpretation is limited by confounding and time-related bias | Baseline IPTW: 48-hour edge contrast at specific time points; MSM: Edge discrete-time contrast applied under control of measured time-varying confounding factors; CCW: Edge compliance scheme contrast calculated based on adherence to a predefined strategy within a 24-hour grace period. |
| Confounding control | Randomization balances measured and unmeasured baseline factors in expectation | Conventional regression or baseline propensity-score adjustment, usually limited to measured baseline covariates | Stabilized IPTW using 14 baseline covariates; MSM employs time-varying weights; the revised CCW constructs a stabilized IPCW using baseline information. |
| Handling of time-varying confounding | Not required for baseline randomization, unless post-randomization adherence or dynamic strategies are analyzed | Often inadequately handled; time-updated clinical variables may act as both predictors of treatment and consequences of prior treatment | MSM incorporated daily CRRT status and lagged urine output, MAP, and vasopressor use to address treatment-confounder feedback |
| Protocol deviation and adherence | Defined according to assigned treatment strategy and grace period | Usually not defined; observed treatment is treated as exposure | CCW cloned each patient into both strategies and artificially censored clones when they deviated from the assigned strategy |
| Missing data | Managed according to a prespecified statistical analysis plan | Complete-case analysis or single imputation may be used | Multiple imputation by chained equations with predictive mean matching was performed using 20 imputed datasets |
| Sensitivity analyses | Prespecified sensitivity analyses for adherence, competing events, and alternative definitions | Often limited to alternative covariate adjustment or subgroup analyses | Exclusion of potential terminal discontinuation; alternative residual-time thresholds; E-value analysis; phenotype-restricted landmark analysis |
| Additional diagnostic objective | Not applicable; the trial estimates the effect of assigned strategies | Rarely assessed; the stability of the exposure construct is usually assumed | Phenotype decomposition is used to assess whether the composition of the clinical population corresponding to the "early discontinuation" label undergoes systematic changes across different landmarks. |
| Follow-up | From time zero to death, censoring, or 30 days | Variable and potentially misaligned with exposure definition | From the 48-hour landmark to death, censoring, or 30 days |

Abbreviations: AKI, acute kidney injury; CCW, clone-censor-weighting; CRRT, continuous renal replacement therapy; ESRD, end-stage renal disease; ICU, intensive care unit; ICD, International Classification of Diseases; IPCW, inverse probability of censoring weighting; IPTW, inverse probability of treatment weighting; MAP, mean arterial pressure; MICE, multiple imputation by chained equations; MSM, marginal structural model; PMM, predictive mean matching; RRT, renal replacement therapy.

Note: The hypothetical randomized trial represents the conceptual target. A conventional observational cohort usually compares observed CRRT discontinuation times without fully aligning eligibility, time zero, treatment strategies, and follow-up. The present study used target-trial emulation to align these components as closely as possible and further evaluated whether “early CRRT discontinuation” was a stable treatment exposure across different landmark times.

**Supplementary Table S2. Missing Data Pattern**

| **Variable** | **Missing, n** | **Missing, %** | **Clinical Reason** |
| --- | --- | --- | --- |
| Urine slope (72h) | 536 | 27.4% | No urine records within 72h window due to death or loss to follow-up |
| Urine slope (48h) | 438 | 25.6% | Incomplete urine records within ±48h of time zero |
| Creatinine slope (d7) | 347 | 24.2% | Death or discharge before day 7 |
| Vasopressor weaning (d3) | 314 | 21.9% | Medication records missing within the specified time window |
| Vasopressor weaning (d7) | 314 | 21.9% | Same as above |
| Lactate clearance (72h) | 241 | 16.8% | Reduced laboratory testing frequency at 72h |
| Urine output (48h total) | 190 | 13.3% | No outputevents records within 24h before time zero |
| Creatinine slope (d3) | 105 | 7.3% | Insufficient creatinine measurements within the 3-day window |
| Fluid overload % (d2) | 40 | 2.8% | Missing weight or fluid balance data |
| Lactate clearance (48h) | 29 | 2.0% | Insufficient lactate measurements around time zero |
| Age, sex, comorbidities, SOFA, MAP | 0 | 0% | — |

Table S2 Note: Complete-case analysis was infeasible (0/1,432 patients with complete observations on all variables). Missing data were handled using multiple imputation by chained equations (MICE) with predictive mean matching, generating 20 imputed datasets (m = 20). The imputation model included all covariates, the exposure variable, the outcome variable, and the Nelson-Aalen cumulative hazard estimator. Abbreviations: SOFA, Sequential Organ Failure Assessment; MAP, mean arterial pressure; MICE, multiple imputation by chained equations.

**Supplementary Table S2A. Window-specific missingness of lagged time-varying covariates used in the MSM**

| Window | Lagged urine missing | Lagged MAP missing | Lagged vasopressor missing | All 3 complete | No daily record vs 48-h cohort |
| --- | --- | --- | --- | --- | --- |
| 48 h | 468/1404 (33.3%) | 0/1404 (0%) | 0/1404 (0%) | 936/1404 (66.7%) | 28/1432 (2.0%) |
| 72 h | 544/1339 (40.6%) | 7/1339 (0.5%) | 0/1339 (0%) | 795/1339 (59.4%) | 93/1432 (6.5%) |
| 96 h | 573/1268 (45.2%) | 31/1268 (2.4%) | 0/1268 (0%) | 695/1268 (54.8%) | 164/1432 (11.5%) |
| 120 h | 623/1209 (51.5%) | 72/1209 (6.0%) | 0/1209 (0%) | 586/1209 (48.5%) | 223/1432 (15.6%) |

Table note：The first three missing rate calculations use the number of person-days for which daily-level records exist within the given time window as the denominator; the "No daily record" metric uses a 48-hour landmark cohort (N = 1,432) as a reference point to reflect the proportion of cases in which the entire daily-level record becomes unobservable thereafter. This latter scenario may be related to events such as death, discharge, termination of CRRT, cessation of monitoring, or data curation and should not be equated entirely with missing data at the project-level. Since the methods for calculating missing values differ between the derived variables for S2A and S2, the numerical values do not need to be consistent.

**Supplementary Table S3.** Causal estimands across analytic approaches

| **Analytic approach** | **Target estimand** | **Effect measure** | **Estimate (95% CI)** | **Interpretation** |
| --- | --- | --- | --- | --- |
| Crude Cox model | Unadjusted association between observed early discontinuation and 30-day mortality among patients alive and on CRRT at the 48-h landmark | HR | 1.15 (0.96–1.38) | Descriptive association without adjustment for measured confounding |
| Baseline IPTW-Cox | Landmark-specific marginal contrast of early versus delayed CRRT discontinuation, adjusted for 14 baseline covariates measured before the 48-h landmark | HR | 1.36 (1.13–1.64) | Estimates the association after balancing measured baseline covariates in a weighted pseudo-population |
| Sequential landmark IPTW-Cox | Landmark-specific marginal contrasts at 48, 72, 96, and 120 h among patients alive and still receiving CRRT at each landmark | HR | 1.13–1.35 | Evaluates whether the exposure-outcome association is stable across changing landmark risk sets |
| RMST (IPTW-weighted) | Difference in mean survival time over 30 days | Days | 3.2 (Delayed – Early) | Provides an intuitive, model-free summary of the survival experience over a fixed time horizon without proportional hazards assumptions |
| MSM | Marginal discrete-time association between daily CRRT status and daily mortality risk, adjusted for lagged time-varying urine output, MAP, and vasopressor use | OR | 1.16 (0.82–1.63) | Addresses treatment-confounder feedback using time-varying inverse probability weights |
| CCW | Marginal per-protocol contrast of adherence to early versus delayed CRRT discontinuation strategies within the 24-h grace period, accounting for artificial censoring | HR | 1.08 (0.91–1.28) | Compares prespecified discontinuation strategies under the revised grace-period CCW framework |

**Note:** Each approach targets a distinct estimand. The estimates should therefore be interpreted as complementary evidence rather than repeated estimates of a single identical causal effect. The MSM reports a discrete-time odds ratio from pooled logistic regression, whereas Cox and CCW models report hazard ratios.

Abbreviations: CCW, clone-censor-weighting; CI, confidence interval; CRRT, continuous renal replacement therapy; HR, hazard ratio; IPTW, inverse probability of treatment weighting; MAP, mean arterial pressure; MSM, marginal structural model; OR, odds ratio;RMST = restricted mean survival time.

**Supplementary Table S4. Protocol specification for the clone-censor-weight analysis**

| Component | Early discontinuation strategy | Delayed discontinuation strategy |
| --- | --- | --- |
| Strategy definition | Stop CRRT within 24 h after the 48-h landmark | Continue CRRT beyond 24 h after the 48-h landmark |
| Grace period | 0–24 h after the 48-h landmark | 0–24 h after the 48-h landmark |
| Compliance definition | CRRT discontinued within the grace period | CRRT continued beyond the grace period |
| Artificial censoring rule | If CRRT was not discontinued within 24 h, the early-strategy clone was censored at the end of the grace period | If CRRT was discontinued within 24 h, the delayed-strategy clone was censored at the time of discontinuation |
| Death during the grace period | Death before strategy deviation was retained as an observed outcome and was not artificially censored | Death before strategy deviation was retained as an observed outcome and was not artificially censored |
| RRT restart after discontinuation | Not used to define strategy adherence; evaluated as part of secondary and multistate outcomes | Not used to define strategy adherence; evaluated as part of secondary and multistate outcomes |
| Censoring-weight model | Stabilized IPCW estimated from baseline clinical covariates measured before the 48-h landmark; primary analysis truncated at P1/P99 | Same baseline-informed specification |
| Target contrast | Per-protocol effect of adherence to early discontinuation versus delayed continuation | Reference strategy |

Note: The CCW approach involves applying two different strategies to patients with 48-hour landmark-based cloners, allowing a 24-hour grace period, with manual censored data when the treatment pathway deviates from the prescribed strategy; death is treated as an endpoint; RRT restart is not used to define adherence to the initial strategy. The primary CCW method utilizes baseline information to construct a stabilized IPCW, with truncation at P1/P99; the weight distribution and alternative truncation schemes are provided in Table S4A.

### Supplementary Table S4A. Diagnostics of stabilized censoring weights in the CCW analysis

| **Diagnostic** | **Value** |
| --- | --- |
| Mean weight | 0.999 |
| Median weight | 0.944 |
| Mean 1st percentile | 0.460 |
| Mean 99th percentile | 2.070 |
| Approximate minimum | 0.459 |
| Approximate maximum | 2.072 |
| Proportion truncated at P1/P99 | 2.1% |
| Mean effective sample size | 1,262 |
| Post-grace-period analytic N | 1,351 |

**Table note:**

Values summarize the distribution of stabilized inverse probability of censoring weights used in the clone-censor-weighting (CCW) analysis across the 20 multiply imputed datasets. The reported 1st and 99th percentiles and effective sample size represent averages across imputations. In the primary CCW analysis, weights were truncated at the 1st and 99th percentiles. The post-grace-period analytic sample comprised patients who contributed follow-up information after application of the 24-hour grace-period strategy. The relatively narrow weight distribution and limited proportion of truncated weights indicated no evidence that the primary CCW estimate was dominated by a small number of extreme censoring weights. CCW, clone-censor-weighting.

Supplementary Table S4B. Sensitivity of the CCW Estimate to Alternative Weight-Truncation Thresholds

| **Weight truncation** | **HR** | **95% CI** |
| --- | --- | --- |
| None | 1.09 | 0.92–1.30 |
| 0.5th/99.5th | 1.08 | 0.91–1.29 |
| 1st/99th | 1.08 | 0.91–1.28 |
| 2.5th/97.5th | 1.07 | 0.90–1.26 |

**Table note:**

Hazard ratios compare the early-discontinuation strategy with the delayed-discontinuation strategy in the CCW analysis. The 1st/99th-percentile truncation was prespecified for the primary analysis; the untruncated, 0.5th/99.5th-percentile, and 2.5th/97.5th-percentile analyses were conducted as sensitivity analyses. Estimates were highly similar across truncation schemes (HR range, 1.07–1.09), indicating that the CCW result was not materially dependent on the selected truncation threshold. Confidence intervals were derived from weighted Cox models with patient-level robust standard errors. CCW, clone-censor-weighting; CI, confidence interval; HR, hazard ratio.

**Supplementary Table S5.** Results of multi-Landmark drift analysis

| **Landmark** | **N** | **Number of Events** | **HR** | **95%CI** | **P value** |
| --- | --- | --- | --- | --- | --- |
| 48h | 1,432 | 679 | 1.32 | 1.10-1.59 | 0.003 |
| 72h | 1,063 | 501 | 1.14 | 0.91-1.42 | 0.25 |
| 96h | 788 | 378 | 1.35 | 1.05-1.74 | 0.02 |
| 120h | 595 | 280 | 1.13 | 0.84-1.52 | 0.81 |

**Table Note:**The four landmarks employed the same IPTW-Cox specification and the same completed dataset to ensure methodological consistency; consequently, the 48-h HR of 1.32 differs slightly from the primary MI-IPTW HR of 1.36. As the landmark is shifted backward, the risk set and the number of events decrease, leading to a significant reduction in the precision of the 120-h HR (1.13; 95% CI 0.84–1.52; P = 0.42). A global interaction P-value> 0.05 merely indicates that no statistically significant interaction was detected and should not be interpreted as evidence of treatment effect homogeneity.

**Supplementary Table S6.** Clinical characteristics (phenotypic breakdown) of patients in the "early withdrawal" group at each Landmark time point

Panel A. Landmark-specific baseline characteristics of patients classified as early discontinuation

| Landmark | N | Age | SOFA | MAP | Creatinine | Urine (mL) | Sepsis (%) | Vasopressor (%) | Ventilation (%) | Liver Disease (%) |
| --- | --- | --- | --- | --- | --- | --- | --- | --- | --- | --- |
| 48h | 369 | 60.1 | 11.4 | 57.6 | 1.84 | 818 | 55.6 | 55.8 | 87.5 | 37.4 |
| 72h | 275 | 61.2 | 11.7 | 57.4 | 1.77 | 888 | 57.8 | 67.2 | 86.9 | 34.9 |
| 96h | 193 | 61.4 | 11.7 | 57.7 | 1.79 | 653 | 64.2 | 72.0 | 90.2 | 37.8 |
| 120h | 139 | 59.3 | 12.3 | 58.6 | 1.91 | 524 | 69.1 | 71.9 | 89.2 | 36.0 |

**Table Note:** Data are presented as mean values or percentages. Baseline clinical characteristics of patients classified as "early discontinuation" (residual CRRT ≤ 24 h) at each Landmark time point are shown. These systematic trends indicate substantial differences among patient populations defined by the "early discontinuation" label at different Landmark time points. Abbreviations: SOFA, Sequential Organ Failure Assessment; MAP, Mean Arterial Pressure.

**Panel B. Formal trend analyses**

| **Characteristic** | **OR per 24-h later landmark** | **95% CI** | **P value** | **Characteristic** | **OR per 24-h later landmark** | **95% CI** | **P value** |
| --- | --- | --- | --- | --- | --- | --- | --- |
| Baseline sepsis | 1.21 | 1.07–1.37 | .002 | Baseline sepsis | 1.21 | 1.07–1.37 | .002 |
| Baseline vasopressor exposure | 1.32 | 1.16–1.50 | <.001 | Baseline vasopressor exposure | 1.32 | 1.16–1.50 | <.001 |

**Table Note：**

Data represent baseline clinical characteristics of patients classified as early CRRT discontinuation (residual CRRT duration ≤24 hours) separately at each landmark. Because the early-discontinuation group was redefined at each landmark, differences in these characteristics reflect changes in the composition of the patient population captured by the exposure definition rather than within-patient longitudinal changes. Landmark time was coded in 24-hour increments for formal trend analyses. Logistic regression was used to estimate ORs and 95% CIs for baseline sepsis and baseline vasopressor exposure.

**Supplementary Table S7. Phenotypic restriction Landmark drift analysis**

| **Landmark** | **Full Cohort** |  |  |  | **Restricted**  **(SOFA 8-14)** |  |  |  |
| --- | --- | --- | --- | --- | --- | --- | --- | --- |
|  | **N** | **Events** | **HR**  **(95% CI)** | **P** | **N** | **Events** | **HR**  **(95% CI)** | **P** |
| 48h | 1,432 | 679 | 1.32 (1.10-1.59) | 0.003 | 868 | 388 | 1.21 (0.95-1.54) | 0.12 |
| 72h | 1,063 | 501 | 1.14 (0.91-1.42) | 0.25 | 645 | 288 | 1.05 (0.78-1.40) | 0.76 |
| 96h | 788 | 378 | 1.35 (1.05-1.74) | 0.02 | 471 | 216 | 1.27 (0.92-1.77) | 0.15 |
| 120h | 595 | 280 | 1.13 (0.84-1.52) | 0.81 | 354 | 162 | 0.98 (0.66-1.45) | 0.92 |

**Table Note:** The restricted cohort was defined as the subgroup of patients with an SOFA score of 8–14. The hazard ratio (HR) represents the risk ratio from IPTW-weighted Cox regression, with early discontinuation of dialysis (residual CRRT ≤24 h) as the exposure variable. The HR range for the full cohort was 1.13–1.35 (Δ = 0.22), while that for the restricted cohort was 0.98–1.27 (Δ = 0.29). No significant reduction in Landmark drift was observed after phenotype restriction. Abbreviations: HR, hazard ratio; CI, confidence interval; SOFA, Sequential Organ Failure Assessment.

**Supplementary Figure S1:** A directed acyclic graph (DAG) illustrating time-varying confounding feedback in the CRRT discontinuation study.

Blue nodes represent the 14 baseline confounding factors (age, gender, heart failure, COPD, diabetes, liver disease, sepsis, SOFA score, minimum MAP, baseline creatinine, 48-hour urine output, 24-hour net fluid balance, mechanical ventilation, and vasopressor use). Orange nodes denote the lagged values (t–1) of time-varying confounding factors, while brown-yellow nodes represent their current values (t). Red nodes indicate exposure variables—the current CRRT status; dark-colored nodes represent outcomes—death; gray arrows indicate causal directions. Blue dashed boxes mark baseline confounding, orange dashed boxes denote time-varying confounding (t–1), and brown-yellow dashed boxes represent time-varying confounding (t). The key causal structure is a treatment-confounding feedback loop: the current CRRT status influences daily urine output, MAP, and vasopressor use, whose current values subsequently affect CRRT decisions the following day (t+1). This cyclic structure cannot be adequately captured by standard regression or baseline propensity score methods and requires correction using time-varying causal inference techniques such as marginal structural models. Abbreviations: DAG – directed acyclic graph; CRRT – continuous renal replacement therapy; SOFA – Sequential Organ Failure Assessment; MAP – mean arterial pressure; COPD – chronic obstructive pulmonary disease.

**Supplementary Figure S2a.** Trace plots for multiple imputation by chained equations. The horizontal axis represents the number of interpolation iterations, while the vertical axis shows interpolation values. Lines of different colors correspond to chains generated by each of the 20 interpolation datasets. All chains exhibit thorough mixing without distinct trends, indicating convergence of the interpolation algorithm; thus, the 20 datasets can be independently utilized for subsequent analyses. The figure illustrates variables with high missing-value proportions in the interpolated datasets (urine volume slope at 48 h and creatinine slope at d3). Abbreviation: MICE (Chain Equation Multiple Interpolation).

**Supplementary Figure S2b.** Density plots comparing observed and imputed values. The blue line represents the density distribution of values generated from 20 interpolated datasets, while the red line shows the density distribution of original observations. Both distributions overlap substantially without systematic deviation, indicating that the interpolated values adequately preserve the distribution characteristics of the original data and that the mean-matching prediction method introduces no additional bias. The variables depicted in this figure are identical to those in Figure S1a. Abbreviation: MICE (Chain Equation Multiple Interpolation).

**Supplementary Figure S3.** Diagnosis of the MSM positivity criterion. The density plot illustrates the predicted treatment probability distributions for patients in the "still receiving CRRT" group (red) and the "has discontinued CRRT" group (green). There is significant overlap between the two distributions (the discontinued group is concentrated at low probabilities, while the CRRT group exhibits a broader distribution), indicating no definitive treatment assignment (i.e., no patients receive 100% or 0% CRRT), thereby satisfying the positivity assumption of MSM. Abbreviations: MSM, Marginal Structural Model; CRRT, Continuous Renal Replacement Therapy.

**Supplementary Figure S4.** Multistate model of termination pathways for the CRRT treatment regimen. Nodes represent clinical states, arrows indicate transitions between states, and each pathway is labeled with the frequency and percentage of events. Among the 1,432 patients who initiated CRRT therapy, 448 (31.3%) discontinued CRRT treatment within 30 days. Of these, 159 cases (35.5%) achieved successful remission (no restart of RRT within 72 hours and survival up to 30 days), 129 cases (28.8%) resumed RRT within 72 hours, and 160 cases (35.7%) died after treatment discontinuation; the remaining 984 cases (68.7%) never discontinued CRRT, among whom 472 (48.0%) died during treatment and 512 (52.0%) were still receiving CRRT at 30 days. This model demonstrates that CRRT discontinuation represents an intermediate clinical state rather than a definitive endpoint. Abbreviations: CRRT – Continuous Renal Replacement Therapy; RRT – Renal Replacement Therapy.

**Supplementary Figure S5a.** Diagnosis of covariate balance before and after IPTW weighting. The horizontal axis represents standardized mean difference (SMD), with each point corresponding to a baseline covariate. Red points indicate the unadjusted SMD before IPTW weighting, while blue points represent the adjusted SMD after weighting. The vertical dashed line marks the conventional balance threshold of SMD = 0.1. Prior to weighting, multiple covariates exceeded this threshold (SOFA SMD = 0.24, inotropic agents SMD = 0.15, sepsis SMD = 0.10); after weighting, all 14 covariates exhibited SMD values <0.1, demonstrating successful intergroup balance achieved by IPTW. Abbreviations: IPTW – inverse probability of treatment weighting; SMD – standardized mean difference; SOFA – Sequential Organ Failure Assessment.

**Supplementary Figure S5b.** Stable IPTW weight distribution. The histogram illustrates the stable IPTW weight distribution across 1,432 patients. A vertical dashed line marks the weight value of 1.0. The weights are centered at 1.0 (mean: 1.00; range: 0.60–1.61), with a symmetric distribution and no outliers, indicating a well-designed propensity score model free from significant positive conditioning violations. Abbreviation: IPTW (Inverse Probability Treatment Weighting).

**Supplementary Figure S6.** Landmark drift validation after phenotypic restriction. The red line represents the hazard ratio (HR) for the entire cohort (N=1,432) at each Landmark time point; the blue line shows the HR for the subgroup with SOFA scores of 8–14 (N=868). The translucent band indicates the 95% confidence interval; the horizontal dashed line marks HR=1. The HR range in the restricted subgroup was 0.98–1.27 (Δ=0.29), similar to that of the entire cohort (1.13–1.35, Δ=0.22). Landmark drift did not significantly diminish after restricting for phenotypic homogeneity, suggesting that exposure variability cannot be entirely attributed to observable phenotypic variation. Abbreviations: HR, hazard ratio; SOFA, Sequential Organ Failure Assessment.

**Supplementary Figure S7.** Landmark-dependent variation under alternative exposure-definition thresholds.

The four lines show hazard ratios (HRs) across the 48-, 72-, 96-, and 120-hour landmarks when early CRRT discontinuation was defined using residual CRRT durations of ≤12, ≤24, ≤36, and ≤48 hours. The horizontal dashed line indicates HR = 1. Across all four definitions, effect estimates varied across landmarks without a consistent monotonic pattern. The HR ranges were 0.93–1.36 for the ≤12-hour definition, 1.13–1.35 for ≤24 hours, 1.06–1.22 for ≤36 hours, and 1.04–1.26 for ≤48 hours. These findings indicate that the observed landmark-dependent variation was not explained solely by the choice of the 24-hour cutoff. Abbreviations: HR, hazard ratio; CRRT, continuous renal replacement therapy.

**Supplementary Figure S8.** Trajectories of physiological parameters stratified by early versus delayed discontinuation of CRRT. The horizontal axis represents time after initiation of CRRT (hours), with dashed lines marking the start time of CRRT and dotted lines indicating the 48-hour Landmark point. Each plot displays mean values ± standard errors for urine output (mL/6 h), creatinine (mg/dL), and lactate (mmol/L). The early discontinuation group (red) exhibited a urine output of 152.6 mL/6 h at 24 hours post-Landmark (discontinuation window), compared to 99.3 mL/6 h in the delayed group (blue); by 96 hours, the early group reached 305.2 mL/6 h versus 136.3 mL/6 h in the delayed group. Lactate levels at the Landmark point were 3.07 mmol/L and 3.54 mmol/L, respectively. The differences in recovery trajectories between the two groups suggest that these trajectory differences suggest that discontinuation timing may partly reflect pre-existing physiological recovery and should not be interpreted as a homogeneous treatment intervention.
